# Onion anthocyanins: Extraction, stability, bioavailability, dietary effect, and health implications

**DOI:** 10.3389/fnut.2022.917617

**Published:** 2022-07-27

**Authors:** Mahesh Kumar Samota, Madhvi Sharma, Kulwinder Kaur, Sarita, Dinesh Kumar Yadav, Abhay K. Pandey, Yamini Tak, Mandeep Rawat, Julie Thakur, Heena Rani

**Affiliations:** ^1^Horticulture Crop Processing (HCP) Division, ICAR-Central Institute of Post-Harvest Engineering & Technology (CIPHET), Punjab, India; ^2^Post Graduate Department of Biotechnology, Khalsa College, Amritsar, Punjab, India; ^3^Department of Processing and Food Engineering, Punjab Agricultural University, Ludhiana, Punjab, India; ^4^College of Agriculture, Agriculture University, Jodhpur, Rajasthan, India; ^5^Division of Environmental Soil Science, ICAR-Indian Institute of Soil Science (IISS), Bhopal, MP, India; ^6^Department of Mycology and Microbiology, Tea Research Association-North Bengal Regional R & D Center, Nagrakata, West Bengal, India; ^7^Agricultural Research Station (ARS), Agriculture University, Kota, Rajasthan, India; ^8^Department of Horticulture, G.B. Pant University of Agriculture and Technology, Pantnagar, Uttarakhand, India; ^9^Department of Botany, Bhaskaracharya College of Applied Sciences, University of Delhi, New Delhi, India; ^10^Department of Biochemistry, Punjab Agricultural University, Ludhiana, Punjab, India

**Keywords:** anthocyanins, bioavailability, dietary effects, extraction techniques, health effects

## Abstract

Anthocyanins are high-value compounds, and their use as functional foods and their natural colorant have potential health benefits. Anthocyanins seem to possess antioxidant properties, which help prevent neuronal diseases and thereby exhibit anti-inflammatory, chemotherapeutic, cardioprotective, hepatoprotective, and neuroprotective activities. They also show different therapeutic effects against various chronic diseases. Anthocyanins are present in high concentrations in onion. In recent years, although both conventional and improved methods have been used for extraction of anthocyanins, nowadays, improved methods are of great importance because of their higher yield and stability of anthocyanins. In this review, we compile anthocyanins and their derivatives found in onion and the factors affecting their stability. We also analyze different extraction techniques of anthocyanins. From this point of view, it is very important to be precisely aware of the impact that each parameter has on the stability and subsequently potentiate its bioavailability or beneficial health effects. We present up-to-date information on bioavailability, dietary effects, and health implications of anthocyanins such as antioxidant, antidiabetic, anticancerous, antiobesity, cardioprotective, and hepatoprotective activities.

## Graphical Abstract

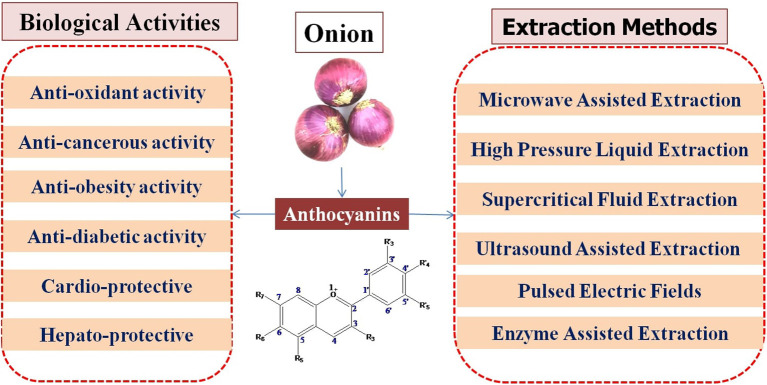

## Introduction

Anthocyanins are a class of plant phenolic pigments and dietary compounds that have a role in human diseases, and these water-soluble pigments are the largest group of plant pigments, contributing different colors, such as the purple, red, and blue, present in fruits, flowers, vegetables, and grains (1). Currently, more people are health-conscious of what they are eating, and as a result, public demand for synthetic pigments such as indigo carmine, Alura Red, and brilliant blue have decreased. Even the food processing and regulatory authorities are seeking to minimize the use of synthetic food dyes and colorants. Nowadays, extensive research focuses on natural colorants like anthocyanins and their health benefits in functional foods (2). Anthocyanins, beyond their bioactivity, are used in foods as natural colorants, fulfilling the public demand for clean labels in food products (3). Anthocyanins seem to possess antioxidant properties, which help prevent neuronal diseases and thereby exhibit anti-inflammatory, cardioprotective, chemotherapeutic, hepatoprotective, and neuroprotective activities, as well as therapeutic effects against other human diseases (4, 5). Because of various health and other benefits, anthocyanins have been accepted by most people as a feed ingredient in recent years. In addition, as a phytonutrient, anthocyanins have anti-mutation and antioxidant activities are important to human health (6). In nature, more than 600 structurally distinct anthocyanins have been identified and characterized (7). For a long time, for enhancing aesthetics and appearance, synthetic colorants have been used. However, because of regulatory concerns, decreasing demands for synthetic colorants, and increasing demands for natural colorants, the food industry and research have shifted toward natural alternatives (8, 9).

Onions are the most important and frequently cultivated vegetable in India as well as worldwide, and the color of the bulb due to flavonoid compounds is an economically important trait. Red- and white-colored bulbs are used for cooking and salad, respectively (10–12). Onion has diverse phytochemicals, such as anthocyanins, flavonoids, phenolic compounds, triterpenoids, and organosulfur compounds, and due to these compounds, it has antioxidant, antibacterial, antidiabetic, and anti-inflammatory activities (13–17). Anthocyanins are the flavonoid compounds responsible for the purple/red color of onion and are highly concentrated in the skin (18). The onion waste is also a rich source of anthocyanins and is reused in foods as bioactive ingredients (19). In red onion, different types of anthocyanins, such as cyanidin 3-laminariobioside, cyanidin mono- and diglucosides, petunidin glucoside, peonidin mono- and diglucosides, and 5-carboxypyranocyanidin 3-glucoside, have been reported (20). Dietary flavonoids found in onions play an important role in human health and nutrition, and studies have reported that onions display many activities including anticancer (21–23), antibacterial (24), hepatoprotective (25), antioxidant (26, 27), antiplatelet (28), immunoprotective (29), anti-cholelithogenic (30), antithrombotic (31), anti-inflammatory (32), and neuroprotective (33) properties. Onion wastes also exhibit inhibitory activity against oxidative stress and enzymes responsible for metabolic syndrome (34–36). Due to their high health benefits to humans, the anthocyanin content of red onion bulbs and skin is extracted during processing (37).

For anthocyanins, different extraction processes have been used, while nowadays, improved methods are used to obtain high yield and improved stability. Anthocyanins are unstable, so it needs procedure optimization to prevent oxidation. Various factors such as temperature, UV radiation, enzymes, pH, chelating metal ion, SO_2_, and ascorbic acid affect its stability, resulting in degradation and color change (38–42).

Therefore, the present review is aimed to deliver a synthesis method from the literature that discusses anthocyanins found in onions, their stability, and extraction technologies that preserve the anthocyanin content. Furthermore, we also discuss the application of anthocyanins in foods and their various implications on human health.

## Anthocyanins found in onion and their stability

**Figure 1:**
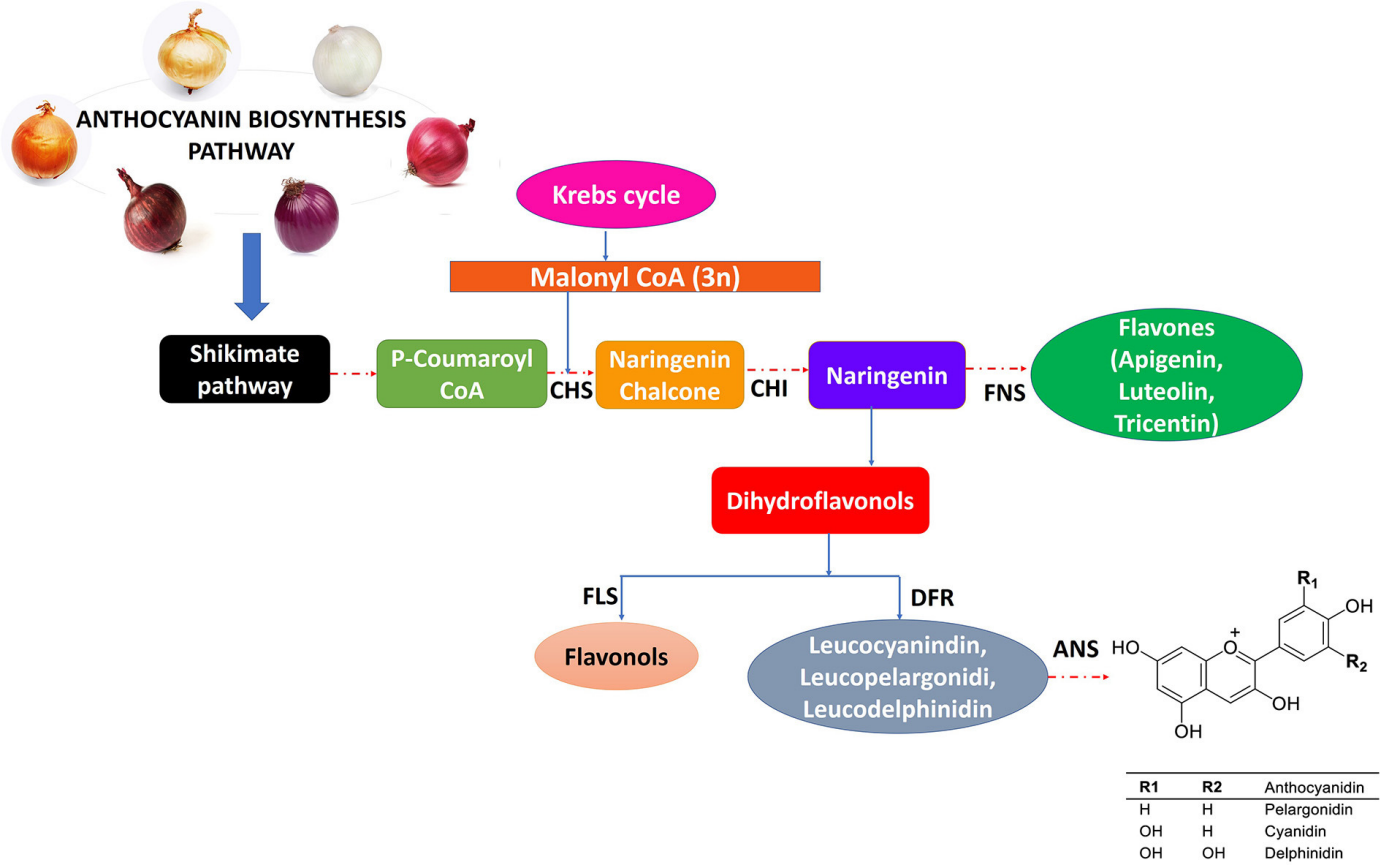
Variety of onion colors and anthocyanins biosynthesis pathway regulated by different enzymes.

In many fruits and vegetables (including onion bulbs), flavonoids and anthocyanins, which are secondary metabolites, are responsible for their vivid colors. Researchers have recently been paying attention to phytochemicals because of their antioxidant properties (43–45). One of their best-described properties is their ability to inhibit free radicals created by cells or environmental factors (44). Despite this, the outermost layers of onions are often discarded while used in raw or in cooked forms, thereby losing a valuable antioxidant component. Onion bulbs and skin contain many bioactive substances, such as fructo-oligosaccharides (FOSs), organosulfur compounds (OSCs), thiosulfinates, polyphenols, and flavonoids (46–48). There are a variety of colors of onions, such as white, yellow, red, pink, orange, and gold (Figure 1), which are primarily due to the presence of two kinds of flavonoids, namely, anthocyanin and flavonols. Anthocyanin generates a wide diversity of colors ranging from red and orange to blue and violet, and flavonols like quercetin and its derivatives give yellow and brown colors to develop in the epidermal cells of scale leaves.

Anthocyanins and flavonoids have distinct chemical structures, react with powerful free radicals, and possess antioxidants, anti-inflammatory, and anticancer properties and Alzheimer's and Parkinson's disease prevention properties (49–51). Research has been conducted on anthocyanins present in onions, leading to the identification of about 10 different types of anthocyanins. Among them, (3-(3-glucosyl-6-malonylglucoside), 3-(6”-malonylglucoside), 3-(3”-glucosylglucoside), and 3-glucoside of cyanidin) are primary anthocyanins (52). White onions have less anthocyanin than red onions. This may have resulted from anthocyanin contents found in each layer of onion. These facts show that red onions are a good source of anthocyanins (50). Among 25 anthocyanins found in red onions, cyanidin 3-O-glucoside is the primary anthocyanin present in the epidermal cells. However, derivatives of cyanidin and peonidin have also been reported. Approximately half of the red onion bulbs contain anthocyanins in the outer epidermis, but the inner epidermis contains smaller amounts (53). Despite being concentrated in the shell or skin, anthocyanin pigments are not present much in the edible portion of red onions. On the other hand, the highest concentrations of anthocyanins were found in the dry skin of red onions, which varied from 109 to 219 mg/100 g on average (50). In contrast to red onion, the white onion skin was reported to contain the lowest content of anthocyanin (0.75 mg/100 g), followed by yellow onion (9.64 mg/100 g) (54). Cyanidin 3-(6-malonylglucoside) (20.95 ± 0.60 mg/kg FW) is detected as major anthocyanin present in bulbs of red onion (*Allium cepa* L.) landrace “Krishnapuram” (KP) (55). The total anthocyanin content reported in onion was (28.6 mg/kg FW), and that of cyanidin 3-glucoside was 1.6 mg/kg FW) (56). Likewise, in Montoro ecotype, the cyanidin 3-glucoside content was 1.19 mg/kg FW (57). The different types of anthocyanins and their derivatives found in different types of onion are presented in Table 1, along with their various detection techniques.

**Table 1:** Different anthocyanin concentrations present in onions and their detection techniques.

**Anthocyanins and its derivative**	**Part used**	Detection technique*	**Concentration**	**References**
Anthocyanins (cyanidin, peonidin, pelargonidin, delphinidin, and petunidin)	Bulb	HPLC	0.3–0.19 mg/100 g DW	(58)
Anthocyanins (cyanidin, peonidin, pelargonidin, delphinidin, and petunidin)	Bulb	HPLC	1,555–250 mg/kg DW	(59)
Anthocyanin	Peel	pH differential	21.99 mg/g DW	(60)
Anthocyanin	Red onion wastes	pH differential	748–840 mg/100 g	(61)
Total anthocyanin	Red, yellow, and white onion	HPLC/DAD-ESI/MS	(29.99 ± 1.19), (9.64 ± 1.30), and (0.75 ± 0.40) mg 100 g−1	(50)
Anthocyanin (cyanidin, peonidin)	Honeysuckle (HSRO) and sweet Italian red onion (SIRO)	LC-ESI-QTOF-MS	HSRO- 0.103 mg/g SIRO- 0.086 mg/g	(18)
Anthocyanin (cyanidin)	Red onions	UHPLC	0.056 mg/g	(62)
Total anthocyanin	Korean red onions	Colourimetric method	0.02–0.12 mg/g	(63)
Anthocyanin (Cyanidin and delphinidin)	Red and white onion	Colourimetric method	Red onion-0.3587 mg/g White onion- 0.014 mg/g	(64)
Cyanidin, Peonidin, Pelargonidin, Delphinidin, and Petunidin	Bulb	HPLC-MS	0.21–0.45 mg/100 g DW	(65)

*For details, refer to the list of abbreviations.

**Figure 2:**
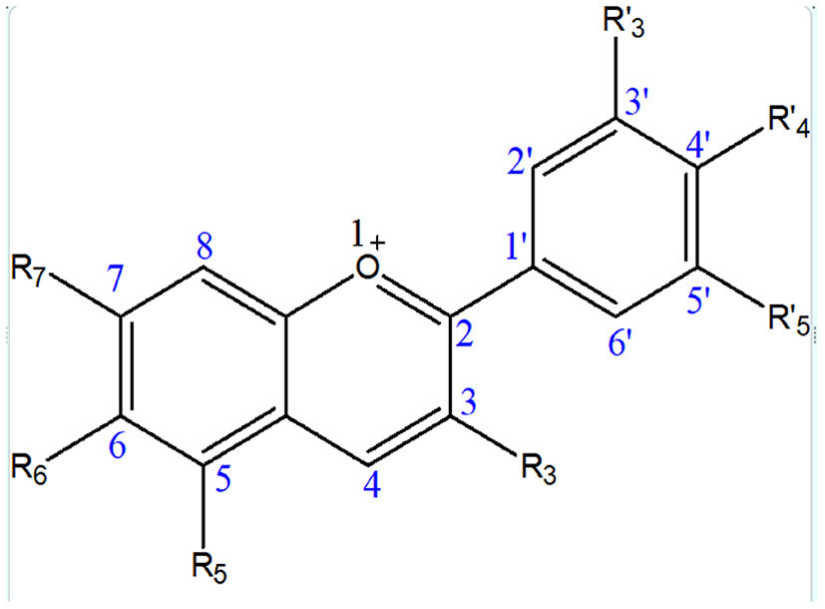
Basic structure of anthocyanins.

**Figure 3:**
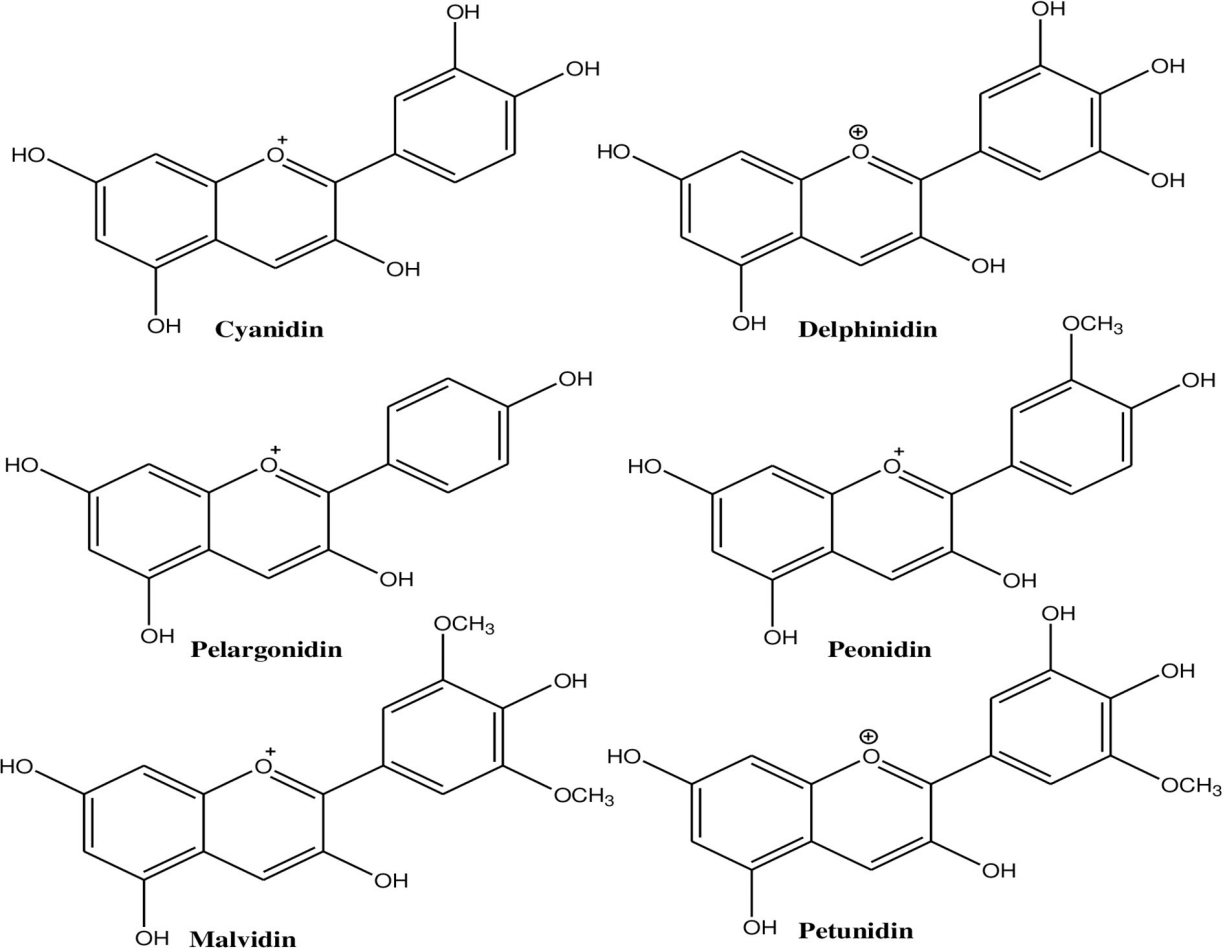
Different types of anthocyanins present in onion that contain -OH or -OCH_3_ groups at different positions.

Anthocyanins contain an anthocyanidin core that consists of a heterocyclic skeleton (Figure 2; called anthocyanidin or aglycon) and -OH or -OCH_3_ groups along with specific sugar or acylated sugar residues primarily at C_3_, C_5_, and/or C_7_ positions, and these can be modified further. It is thought that there are around 20 different core structures, which are named after the plants from which they were isolated: cyanidin, delphinidin, malvidin, pelargonidin, peonidin, and petunidin (Figure 3). As far as onions are concerned, they are frequently reported to contain cyanidin derivatives, as well as a few peonidin, pelargonidin, and delphinidin derivatives (66, 67). According to Bystricka et al., a variety of red onions contain anthocyanin glycosides, including cyanidin, peonidin, and pelargonidin (68). There has been some evidence that the edible portion of red onion contains about 250 mg/kg anthocyanins with cyanidin-3-glucoside as major components (44, 50, 54). The other derivatives of acylated and non-acylated anthocyanins, such as cyanidin mono- and diglucosides, peonidin mono- and diglucosides, petunidin glucoside, cyanidin 3-laminariobioside, and 5-carboxypyranocyanidin 3-glucoside, have been found in red onions (60).

Biosynthesis of anthocyanin occurs as part of a specific section of the flavonoid synthesis pathway, which is controlled on several levels. Through cinnamate-4-hydroxylase (C-4-H) and 4-coumaroyl CoA ligase (4-CL), phenylalanine is converted into cinnamic acid, which then undergoes conversion into 4-coumaryl CoA, an anthocyanin precursor. The next step is the condensation of one 4-coumaroyl CoA molecule and three malonyl CoA molecules using the chalcone synthase, which produces chalcones. In the final stage, a series of enzymatic reactions results in the synthesis of main anthocyanins. As anthocyanins are synthesized in the cytosol, they are transported to the vacuole, where they are stored in the form of color coalescences called anthocyanic vacuolar inclusions (53) (Figure 1). Various transcription factors and regulatory proteins are responsible for flavonoid biosynthesis (67). There is little information available on how flavonoids are regulated in onions. Recently, a report described the role of MYB1 as a transcription factor controlling anthocyanin biosynthesis in onions (69). The researchers also identified putative R2R3MYB genes in onions that are involved in the biosynthesis of anthocyanins and flavonols.

### Factors affecting anthocyanin stability

Varying anthocyanin intensities and stability depend on several factors including the quantity and structure of anthocyanins, pH, temperature, light intensity, nature, and the existence of other pigments in conjunction with metal ions, enzymes, oxygen, sulfur dioxide, ascorbic acid, sugar, sugar metabolites, etc. (70). Based on structural activity studies, it was found that anthocyanins can be stabilized by the modification of their molecules through polymerization, cleavage, and derivatization. Anthocyanins are cleaved to produce colorless compounds, polymerization leads to browning, and derivatization produces colored compounds (71). Stabilizing reactions can be accomplished *via* glycosylation, methylation, and acylation, as well as anthocyanin aglycone synthesis (72). As anthocyanidins are hydroxylated, for instance, delphinidin pigments are shifted toward blue color, whereas when -OCH_3_ groups are glycosylated, red pigments with higher stability are synthesized (52). The stability is also enhanced by the acylation of the sugar moiety with aliphatic or aromatic acids. Numerous aromatic acids can be co-acylated, such as p-coumaric acid, gallic acid, sinapic acid, and ferulic acid, in addition to a wide variety of aliphatic acids, such as malic, tartaric, succinic, oxalic, and acetic acid (72, 73). Despite this, the colors formed by anthocyanins will also be affected by both the vacuolar environment and the structure created by anthocyanins.

#### pH

pH is a major factor that affects anthocyanins. Anthocyanins exist in six different states in the acidic vacuole of the plants. With pH 3 or less, the flavylium cation dominates and creates purple, orange, and red colors as the ionic nature of anthocyanins is found in different forms, which depend on pH. The flavylium form of anthocyanins is found at acidic pH = 1, and the quinoidal form is found at pH between 2 and 4, while the carbinol pseudobase form is found at pH between 5 and 6. Finally, at a pH higher than 7, anthocyanins will be degraded (52, 74–76). It has been shown that decreasing pH 2.8 in anthocyanin can increase the transfer of the structure to the flavylium cation, thereby improving stability; however, changing the pH of a liquid or gel could affect its sensory characteristics (77). It has been found that the color of anthocyanins can change because anthocyanins interact with other pigments and stack to form supermolecular structures, which stabilize at acidic pH of the colored forms and help in interaction with metal ions (53, 78, 79). Also, anthocyanin is much more soluble in water at lower pH as a result of the flavylium cation. Anthocyanins can undergo various acid–base, hydration, and tautomeric reactions when dissolved in water (80). With an increase in pH, the hydration reaction of the flavylium cation becomes more competitive with the proton transfer reactions involving acidic hydroxyl groups (60). In a pH range of 5-6, chalcone and carbinol pseudobase appear, and both are colorless (81). In addition, at a pH higher than 7, anthocyanins undergo degradation, depending on the substituent groups. Due to anthocyanin stability at low pH levels, foods with low pH levels are ideal for using anthocyanins. The presence of hydroxyl or methoxyl groups is responsible for the stability of anthocyanins. In neutral pH, mono- and diglycoside derivatives are more stable, while aglycones are not stable because in mono- and diglycoside derivatives of the sugar moieties will avoid the degradation of anthocyanins (82, 83). In short, the stability of anthocyanins decreases with an increasing number of hydroxyl groups, while it increases with increasing methylation and acylation, thus improving the stability of anthocyanins (84).

#### Temperature

Another important factor that influences the anthocyanin stability is temperature. Heat generally affects the anthocyanin stability through the activation of degradation, causing native enzymes. With an increase in temperature, there are a variety of mechanisms through which anthocyanins can be damaged and lost such as glycosylation, cleavage, nucleophilic attack by water, and polymerization (85). Although anthocyanins are weak substrates for enzymes, it has been demonstrated that B-glucosidase can affect anthocyanin persistence by generating anthocyanidins that can be further oxidized, by polyphenol oxidase (PPO) and/or peroxidase (POD) (86, 87). According to Jeya Krithika et al., anthocyanin in onion peels degrades when the temperature reaches a maximum of 100°C. Anthocyanin also interacts with objects due to its light sensitivity (60). Therefore, light is a major factor that influences the strength of anthocyanin. A recent study by Chithiraikannu et al. revealed that half of the pigments were destroyed when onion peels were placed in darkness for 135 days at 20°C, suggesting that acylation played a protective role (60). It was proven that an enhanced adjustment of the particle of anthocyanin in pH 1 buffer can increase absorbance because of the excitation of flavylium cations. Since anthocyanins are unsaturated molecules, they are susceptible to degradation by oxygen in two different ways: by direct oxidation and by enzyme-mediated decomposition (88). By contrast, some researchers found that phenols and anthocyanins in food increase during the first 7 days of cold storage if it is stored in an oxygen-enriched environment at a low temperature, during the initial days of storage (89). These enzymes cause anthocyanins to lose their solubility and transform them into colorless compounds, thus losing the pigment intensity of color (70). In addition, different plant regions and types of plants exhibit different amounts of anthocyanin depending on the regulatory molecules (precursors and enzymes) of biosynthesis and degradation pathways (52, 70, 90). Due to high temperature, anthocyanins undergo degradation by different mechanisms such as polymerization, glycosylation nucleophilic attack of water, and cleavage (85, 91). Therefore, with the increasing temperature, the degradation of anthocyanins occurs (92). The acylated form of anthocyanins has more stability than the non-acylated form at higher temperatures (93). However, heat treatment for a short period of time inhibits the enzymes and improves the stability of anthocyanins (94, 95) while at the same time prevents the thermal degradation of anthocyanins by decreasing the pH value. Furthermore, the decrease in oxygen concentration prevents the thermal degradation of anthocyanins (96, 97).

## Extraction technology of anthocyanins

**Figure 4:**
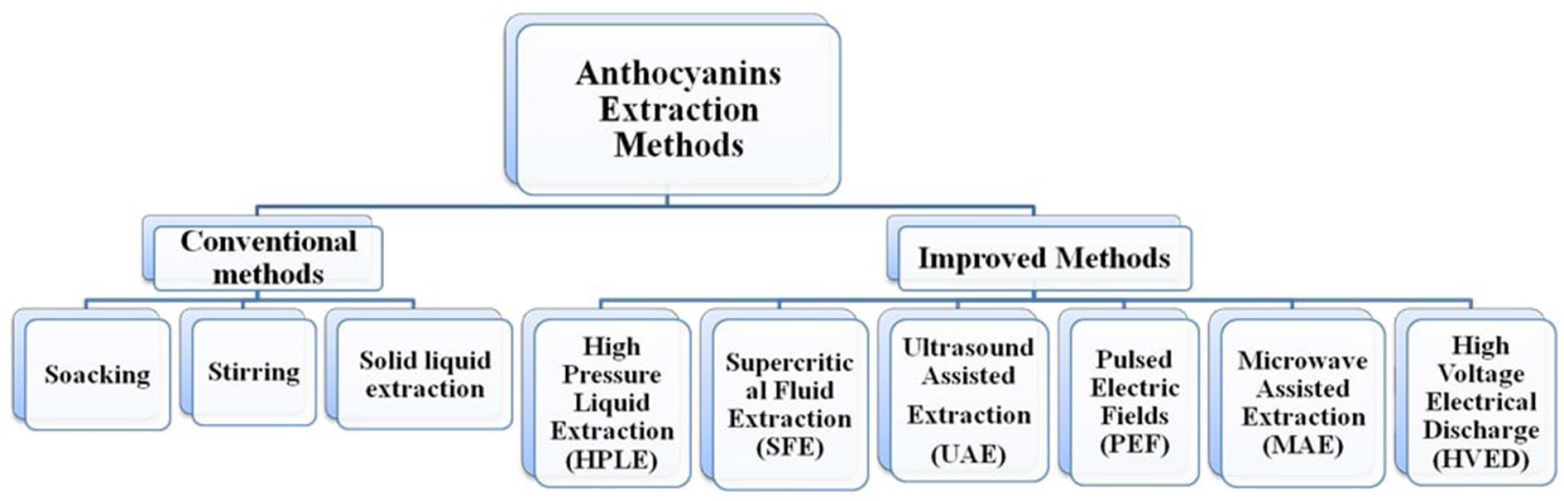
Different anthocyanin extraction methods.

The anthocyanin extraction methods are broadly classified into two groups: first, conventional methods such as maceration, soaking, and heat-assisted extraction, which are easy to use and do not require much instrumentation but have limitations such as the possibility of solvents in the extract, toxicity of solvents, low yield, and thermal deterioration of anthocyanins; second, advanced technologies such as microwave-assisted extraction (MAE), high-pressure liquid extraction (HPLE), supercritical fluid extraction (SFE), ultrasound-assisted extraction (UAE), pulsed electric field extraction (PEFE), enzyme-assisted extraction (EAE), and high-voltage electrical discharge (HVED) (Figure 4), which are sophisticated but have advantages such as high stability of anthocyanins, lower or zero use of solvents, higher yield, and lower energy cost (38, 39, 41, 42, 64, 98–101).

### Conventional methods

Conventional methods depend on the solubilizing capacity of solvents and for anthocyanins, ethanol, water, methanol, acetone, or mixtures thereof used. Anthocyanin is an unstable compound, so for stabilizing, weak acids such as citric acid, formic acid, or acetic acid are added in the solvent because anthocyanins are more stable at acidic pH. In addition to the solvents, the yield of anthocyanin depends on the size of the solid powder, time, temperature, and solvent-to-solid ratio. The anthocyanins are extracted by different polar solvents because of polar characteristics of anthocyanins, and selection of suitable solvents such as acetone, water, methanol alcohol, acidified methanol, or acidified acetonitrile is a crucial step because anthocyanins are highly reactive molecules (102–104). Zhang et al. reported using 0.1% hydrochloric acid in an 8:2 ratio of ethanol and water and found a higher concentration of anthocyanins and antioxidant activities in red, yellow, and white onions (50). Using the acidified ethanol and water, it was possible to extract a higher anthocyanin content of 22 mg/g dry material (105). Chandrasekhar et al. reported that using 50% (v/v) ethanol in water, the maximum anthocyanin content of 381.1 mg/L was extracted, while the higher anthocyanin content was recovered using acetone (425.9 mg/ L) and 1% HCl in methanol (419.7 mg/L) because of more polar solvents (106). Musso et al. observed that when the extract with water ethanol was stirred for half an hour at 300 rpm, the anthocyanin contents found in the alcoholic and aqueous extracts were 4 and 0.6 mg/L, respectively (107). Galvao et al. also observed that in a magnetic stirred batch with water, methanol, isopropanol, and ethanol at a controlled temperature of 25 C for a period of 3 h, the anthocyanin contents were found to be 50.37, 19.44, 0.77, and 0.76 mg/ L, respectively (108). Stirring in an orbital shaker with acidified water (0.05 M H_3_PO_4_) at 90 rpm for 12 h, the anthocyanin concentration was 32.05 mg/L (109). The conditions were optimized to extract anthocyanins with ethanol (9.1% v/v) acidified to pH 3 using citric acid because in acid pH, stabilization of the anthocyanin structure is high. It is concluded that with mild temperatures, the amounts of anthocyanins increased, while with longer time and high ethanol concentration, it decreased because longer time led to breakdown of cyanidin-3-O-glucoside compounds (110). Backes et al. also observed that using a mixture of ethanol (100% v/v) acidified to pH 3 and citric acid because in acidic pH, the degradation of anthocyanins is less because the structure of anthocyanins is more stable (111). The author revealed the yield of anthocyanin contents increased with the increase in ethanol concentration and temperature.

### Improved methods

To overcome the disadvantages of conventional extraction methods, improved methods have been introduced for extraction. These methods are mainly focused on higher yield, environment-friendly, and industrial use. Among these improved methods are microwave-assisted extraction (MAE), high-pressure liquid extraction (HPLE), supercritical fluid extraction (SFE), ultrasound-assisted extraction (UAE), pulsed electric field extraction (PEFE), enzyme-assisted extraction (EAE), and high-voltage electrical discharge (HVED).

#### Microwave-assisted extraction (MAE)

The MAE method uses radiation in the microwave region such as electromagnetic radiation and to rupture the cell wall (112–114). Thus, the extract easily comes out of the cell to the solvent. Different conditions should be optimized during the MAE process, such as microwave power, liquid-to-solid ratio, time, particle size, and temperature. Xue et al. reported that the highest anthocyanin content was found at a temperature of 50.75°C (115), and Sun et al. also found the lowest degradation of anthocyanins was at a temperature of 53.3°C (116). Xue et al. found that the highest anthocyanin content was observed at 8-s irradiation time and a temperature of 50°C (117). The anthocyanins were found maximum (1,023.39 mg/ kg) at the conditions of a solid-to-water ratio of 1.3 g/ml, 800 W of microwave power, and 8 min of extraction time. These results indicated that reduction in extraction time enhances the extract concentration due to lower degradation of anthocyanins (118). Recently, the same results were also revealed by Nguyen et al., who found that the reduction in extraction time enhances the anthocyanin content. They found the optimal condition for maximum anthocyanin content (73.89 mg/L) was 60% ethanol concentration, 600 W of microwave power, a solid-to-solvent ratio of 1.30, and 2-min extraction time (119). Using ethanol as a solvent (100%; pH 3), optimized anthocyanin extraction conditions were a temperature of 62°C, time of extraction of 300 s, and 100:5 (mL/g) liquid-to-solid ratio. The anthocyanin content found was 411 mg/g of DW, and they also concluded that anthocyanins are more stable at lower pH (111). Farzaneh and Carvalho also optimized the conditions using water as a solvent at a temperature of 50°C, a time for extraction of 114 s, and a 30:1 (mL/g) liquid-to solid-ratio; the anthocyanin content found was 273.3 mg/L (120).

#### Ultrasound-assisted extraction (UAE)

The UAE method is based on ultrasound force that causes cavitation and breaks the cell wall (121, 122). The extraction of anthocyanin in the UAE method depends on different parameters such as temperature, solvent composition, time, the liquid-to-solid ratio, particle size, moisture content in the matrix, pulse cycle, and ultrasound power (110, 113, 123–126). The results obtained show a better yield (32 mg/g) of anthocyanin extracted from jabuticaba (110), which is in line with the results of Backes et al. and Demirdoven et al. that showed a higher yield of anthocyanin obtained by using the UAE method from red cabbage and fig peels, respectively (111, 127). Demirdoven et al. extracted anthocyanins using ethanol, water, and formic acid in an ultrasound bath operating at 37 kHz frequency. They extracted 11.92% more anthocyanins than those extracted using conventional methods at optimal conditions 40°C of temperature, 75 min of extraction time, and 45% of ethanol concentration. So, the anthocyanins were extracted at low temperature and low solvent concentration because at higher temperature, the degradation of anthocyanins is high (127). Ghavidel et al. also optimized the conditions for the extraction of anthocyanins using an ultrasonic power of 35 kHz, and the extracted concentration of anthocyanins was 9.468158 mg/L, with an ethanol-to-hydrochloric acid (15:85) at 60.94°C for 40 min (128). The anthocyanin yield was maximum (20.9 mg/L) using water as a solvent at 30 min of extraction at 15°C and 100 W using the UAE method because higher temperature and longer time of extraction degraded anthocyanins (124, 129, 130). Ravanfar et al. also revealed the optimal condition for extraction using water as a solvent at a different temperature, extraction time, ultrasonic power, and ultrasonic pulse duration for maximum anthocyanin yield (60 mg/L): power of 100 W, 15°C of temperature, 90 min of extraction time, and pulsation mode of 30 s (131). Using ethanol as solvent (34.47%) and anthocyanin extraction conditions optimized at a temperature of 35°C, time for extraction of 24 min, 100:5 (mL/g) liquid-to-solid ratio, the anthocyanin content was 32 mg/g of the extract (110).

#### Pressurized liquid extraction (PLE)

PLE is based on the use of solvents at different temperatures and pressure. The extraction yield of anthocyanins depends on different parameters such as temperature, static time, pressure, and the number of cycles (132–135). Wang et al. reported a higher yield of anthocyanins (8.15 mg/g) at 70 bar and 130°C during 90 min by using the PLE method (136). Kang et al. also found a maximum yield of anthocyanins at 100 bar and 130°C for 3 min from blueberries (74). Another method based on PLE is the high-pressure liquid extraction (HPLE) method, which is based on high pressure and keeps the solvent above its boiling point (100). In the HPLE method, the recovery efficiency mainly depends on the solvent, pressure, temperature, and the solvent-to-solid ratio (41). The optimal condition for maximum recovery of anthocyanins was found at 384 MPa pressure, for 15 min with 35% (v/v) ethanol (137) and increased with time from 15.1 to 39.4% from 5 to 15 min (138). Arapitsas and Turner, extracted anthocyanins with water + ethanol + formic acid in conditions temperature 80–120°C, sample amount 1–3 g, extraction time 6–11 min at a pressure of 50 bar, and anthocyanin yield was 662 μg/g of the sample. They revealed that the best conditions for anthocyanin extraction was at 2.5 g of sample, 7 min of extraction time, 99°C of temperature at 50 bar of pressure, and solvent composition of 94/5/1(v/v/v) of water/ethanol/formic acid (139). Pereira et al. also optimized the conditions for extraction of anthocyanins and found 10.21 mg malvidin-3-O-glucoside/g DW at an ethanol concentration of 50%, reactor volume of 50 mL, extraction time of 220 min, solvent flow of 5 g/min, temperature of 100°C, and pressure of 100 bar (140).

#### Pulsed electric field extraction (PEFE)

PEFE application is based on the increase in the electropermeabilization process to enhance the cell membrane permeability by an electrical force. The recovery of anthocyanins in the PEFE method depends on the electric field strength, the exposition time, initial temperature, and the total specific energy (42, 141–144). Thus, Gagneten et al. observed the effect of the temperature at 10 or 22°C and found higher extraction recovery at 22°C (145). Many studies have observed that recovery of anthocyanins increased with increase in the pulse number and pulse intensity (146–148). In the PEF method using water as a solvent with the conditions as a temperature of 22°C, electric field strength of 2.5 kV/cm, and specific energy of 15.63 J/g, the anthocyanin concentration was 44 to 889 μg/mL. Total anthocyanin extraction increased by 2.12 times because the non-acylated forms of anthocyanins have high proportion than the control (149).

#### Enzyme-assisted extraction (EAE)

In the enzyme-assisted extraction (EAE) method, pectinases, proteases, cellulases, and hemicelluloses have been used to accelerate and enhance the recovery of pigments from different matrices (150, 151). EAE has higher efficiency than the conventional methods for the extraction of pigments, and extraction depends on different conditions such as pH, the composition of the enzymatic mixture, temperature, liquid-to-solid ratio, and time (150–154). In EAE, using pectinex containing pectinase, cellulose, and hemicellulase at pH of 3.5, temperature of 45°C, liquid-to-solid ratio of 10:1, and hydrolysis time of 120 min, the anthocyanin concentration was 675 mg/100 g of the extract (151, 155). Li et al. also optimized the conditions using pectinase enzyme at pH of 5.9, temperature of 45°C, liquid-to-solid ratio of 20:1, and hydrolysis time of 58 min, anthocyanin concentration was 6.04 mg/g of sample (156).

#### Supercritical fluid extraction (SFE)

SFE efficiency depends on different conditions and needs to be optimized during the extraction process, and these conditions are pressure, amount of co-solvent, temperature, particle size, extraction time, moisture content, the flow rate of CO_2_, and the liquid-to-solid ratio (157–164). Maran et al. optimized the conditions for maximum recovery and found a higher yield at 50°C and 2 g/min solvent flow rate (163). Jiao et al. showed a higher yield of anthocyanins and antioxidant activity were obtained by using the SFE method than by using conventional extraction methods (164). Jio and Kermanshahi optimized the extraction conditions for the extraction of anthocyanins with a flow rate of 10 mL/min and found the anthocyanin concentration was 16.7–57.7%, which was more than that obtained using conventional methods (164). Researchers optimized the extraction conditions using acidified water under pressurized CO_2_ at different temperatures, extraction times, and pressure levels and found the optimal conditions at 60°C, 10 Mpa of pressure, and extraction time of 20 min yielded higher anthocyanin contents. They reported that the reduction in extraction time helps in increased efficiency of anthocyanin extractions (165). SFE with CO_2_ and co-solvent water is used for the extraction of anthocyanins. The anthocyanin yield was 25 mg/g of DW at pressure of 450 bar, flow rate of 15 min static time, and 20 min dynamic time at 10 mL/min (164). Maran et al. also optimized the extraction of anthocyanins with ethanol co-solvent, and the anthocyanin concentration found was 231.28 mg/100 g of the raw material at a pressure of 162 bar and a flow rate of 2 g/min (163).

## Applications in foods and health benefits

**Figure 5:**
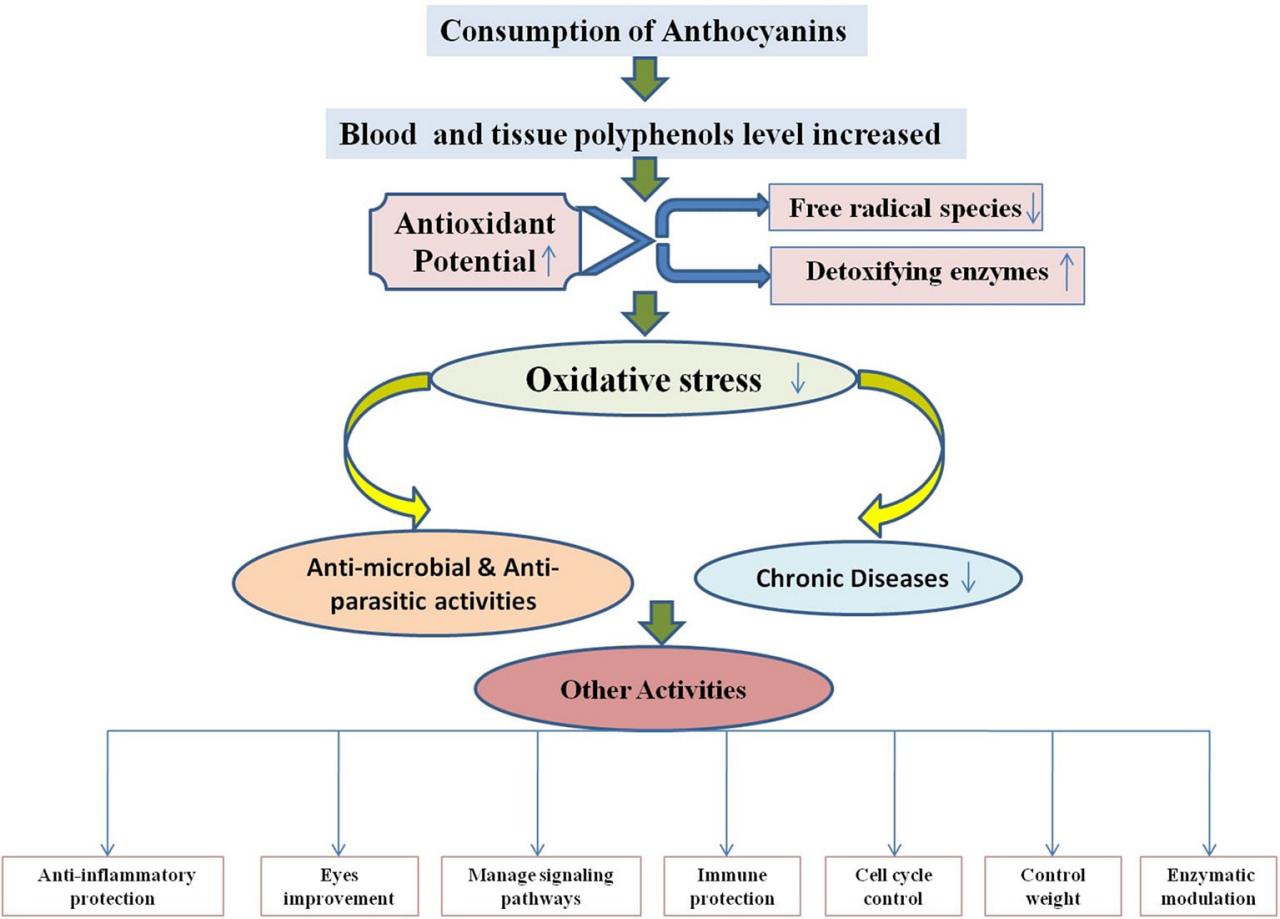
Mechanism of antioxidant potential of anthocyanins and reduction of chronic diseases, microbial, and parasitic activities.

As a necessary consequence, several studies on the delivery of anthocyanins from various sources into foods and their products like beverages, dairy products, soft drinks, jams, confectionery, and more have been carried out in recent years (42, 166, 167). Recent studies on *in vitro* experimental systems using anthocyanin-rich extracts or purified anthocyanins have confirmed high potential to applications in foods. Protection against liver damage, improvement in eye sight, significant reduction in blood pressure, strong anti-inflammatory and antimicrobial activities, and suppression of human cancer cell proliferation are all demonstrable benefits (Figure 5). Also, Figure 5 demonstrates that whenever there is any inflammation in the body or any chronic disease, parasitic infection, or microbial infection, it causes oxidative stress due to release of free radical species in the body. When anthocyanins are used, they act as an antioxidant and neutralize the free radicals and thereby decrease the oxidative stress.

### Bioavailability and dietary effects of anthocyanins

Food extracts high in anthocyanins have been used in the development of dietary food supplements. Anthocyanins extracted from purple corn are used as an antioxidant dietary supplement are good health promoters. Anthocyanin is a bioactive component found in foods used as an appetite stimulant, phyto-pharmaceutical drug, and choleretic agent and for treatment of many other diseases (168). Most studies on the bioavailability of anthocyanins indicate that it is absorbed rapidly following consumption (about 0.22 to 2 h) and that it is excreted within 6 h (169, 170). *In vitro* studies on humans and animals show that anthocyanins are absorbed in their intact glycosidic form, unlike other flavonoids (171–173). Evidence shows that <1% of consumed anthocyanins are detected in the urine or plasma of humans (174–176). The bioavailability of anthocyanin as a nutraceutical is critical for maintaining good health and disease prevention like malvidin-3-glucoside, and cyanidin-3-glucoside is well-documented among major anthocyanins. Peonidin glycosides have the highest relative bioavailability, followed by cyanidin, malvidin, delphinidin, and petunidin glycosides of red wine anthocyanins (177, 178). Recently, anthocyanins are encapsulated with different dietary fibers such as gum arabic (179) or β-cyclodextrin (180) for their low release of anthocyanins during digestion. The encapsulation techniques enhanced the bioavailability of anthocyanins in the colon and delayed the release (181, 182). A study demonstrated the bioavailability and absorption of malvidin-3-glucoside anthocyanins, and it was observed that anthocyanins were found in the plasma and urine after 3 and 6 h of ingestion of red wine and red grape juice, respectively (183). Another finding also observed anthocyanins in the urine of volunteers who consumed 218 mg anthocyanins *via* 300 ml red wine and found that anthocyanins peaked in urine after 6 h of consumption. Recently, through ^13^C-tracer methodology, methylated and sulfo-conjugated metabolites were identified in urine and helped in the identification of degradation products during circulation through urine (184). Anthocyanins help reduce cholesterol levels and marketed as dietary supplements (185). Additionally, blue wheat bran was also used as dietary supplements and processed to produce an anthocyanin-rich blue wheat powder (186).

Anthocyanins are absorbed rapidly in the oral cavity of humans and appeared in the bloodstream after consumption, and degradation started in mouth. Absorption and degradation processes depend on oral microbiota (187). Anthocyanins have the ability to cross the blood–brain barrier and were observed in the endoepithelial cells, but not in certain tissues. Nowadays, different health organization suggested consumption of colorful vegetables and fruits because they provide essential nutrients and bioactive compounds (188, 189).

### Physiological and health implications

**Figure 6:**
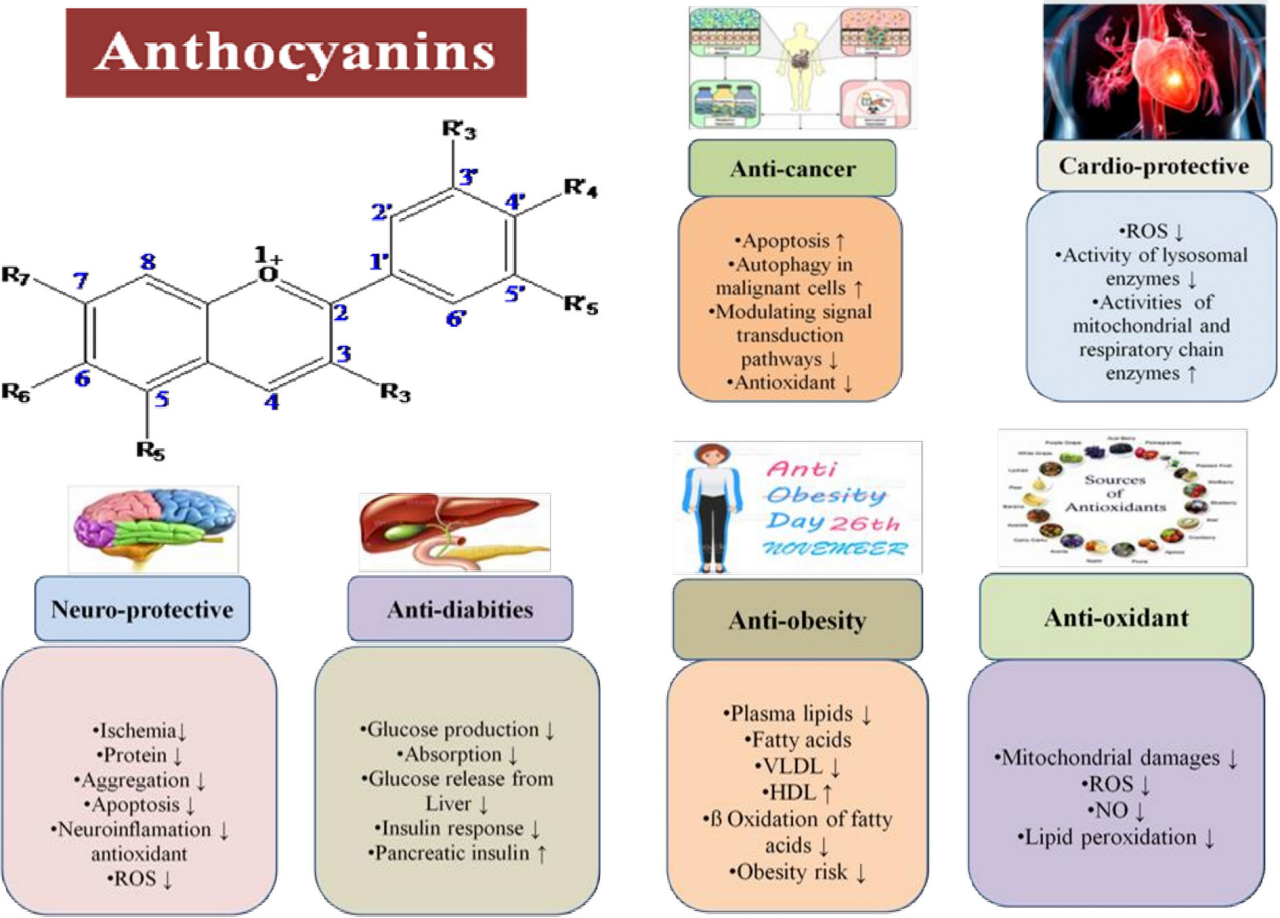
Physiological and health implications of anthocyanins.

There has been much research on anthocyanins that shows their therapeutic and food applications. As previously demonstrated, anthocyanins have a wide range of health and therapeutic effects (Figure 6). Table 2 presents the biological activities of anthocyanins in different systems and their health effects.

**Table 2:** Biological activities of anthocyanins and their health effects.

**Cell culture**, ***in vivo*** **or clinical study**	**Biological activity**	**Health benefits**	**References**
Cell Studies	Serviceable against insulin resistance and diabetes	Anti-diabetic effects	(190)
Oral capsule	Improvement of vision in patients with open-angle glaucoma	Beneficial for eye health	(191)
Primary intravenous in rats	Protective effect during retinal inflammation	Eye health	(192)
Bioassay-directed fractionation	Suppressed cell proliferation, inflammation, and angiogenesis and induced apoptosis in esophageal tissue of rats	Anticancer effects	(193)
Male Wistar rats were fed the anthocyanin-rich (ACN-rich) or the anthocyanin-free (ACN-free) diet	Decreased susceptibility to ischemia-reperfusion injury and infarct size with increased myocardial antioxidant enzyme	Beneficial against Cardiovascular diseases	(194)
Anthocyanins was added to the animals' water bottles every day	Promoted apoptosis in benign prostatic hyperplasia rats	Anticancer effects	(195)
Oral squamous cell	Decreases NF-*k*B1 and PTGS2 activity	Anticancer effects	(196–198)
Carcinoma patients	Increases AURKA, BIRC5, and EGF activity	Anticancer effects	(198)
MDA-MB-453 breast cancer cells	Increases Caspase-3 cleavage activity	Anticancer effects	(199)
Esophageal squamous cancer cell in rats	Decreases COX-2 and iNOS activation of ERK AKT expression	Anticancer effects	(198–200)
Microbial strains	Highest sensitivity to *Aeromonas hydrophila* and *Listeria innocua*	Antimicrobial effects	(201)
Oral capsule	Improvement of dyslipidemia, enhancement of antioxidant capacity, and prevention of insulin resistance in human with type 2 diabete	Antidiabetic effects	(202)
Oral solution	Amelioration of renal apoptosis in diabetic nephropathy mice	Antidiabetic effects	(203)
Fat diet-induced mouse model	Suppression of body weight gain and improve blood lipid profile in rats	Antiobesity effects	(204)
*In vitro* simulated gastroduodenal digestion	Increasing glucose absorption, decreasing glucose diffusion rate and promoting glucose transport across the cell membrane	Antioxidant, hypoglycemic, and hypolipemic effects	(205)
Cell Study	Modulatory effect on the composition and abundance of human intestinal microbiota.	Prebiotic activity	(206)
Cell study	Enhance the high glucose plus palmitic acid induced ROS	Antimicrobial effects	(207)
Cell study	α-glucosidase inhibitory activity and ROS scavenging activities o	Antioxidant activity	(208)
Trolox equivalent antioxidant capacity (TEAC) was measured	Falicitate unique structural features like 4'-glycosylation and unsusual substitution pattern of sugar moities	Antioxidant activity	(44, 209)
HPLC, DPPH (radical-scavenging activity), ORAC methods	Preventing the growth of tumors	Antiproliferative effects	(58, 210, 211)
HPLC	Increase in total flavonoids, total phenolic content, total anthocyanins, protein, and calories	Antioxidant activity	(18)

#### Antioxidant effects

Antioxidants in the diet can help reduce the generation of reactive oxygen species (ROS) and free radicals, lowering the risk of cancers and heart diseases. The antioxidant properties of anthocyanins are superior to well-known antioxidants such as butylated hydroxyl anisole (BHA) (212, 213). In addition to the ability of anthocyanin compounds to act as radical scavengers, the possess the ability to attack 2,2-diphenyl-1-picrylhydrazyl (DPPH) free radicals. DPPH is a stable free radical as the majority of its electrons are distributed over the whole molecule (214, 215). It has been suggested that the differences in DPPH-scavenging capacities of delphinidins and cyanidin-based anthocyanins are due to their molecular structure, which can determine their antioxidant properties and their bioactivity. For example, there are several natural antioxidants, but their amounts are low or they are primarily located in the pericarp and the embryo, which almost get destroyed in the processing (216). Using a genetic engineering technique called biofortification, we can generate different varieties with high anthocyanin contents, and this may help in the prevention of diseases (217). Antioxidant activity has been reported in different onion landraces such as “Cipolla di Giarratana” (218), “Vatikiotiko” (219), “Tropea” (220), and “Bianca di Pompei” (221). Vijayalakshmi et al. reported that onion extracts protect erythrocytes effectively (23%), against oxidative damage induced by hypochlorous acid in human erythrocytes (55), and Tedesco et al. also found onion extract inhibits hemolysis of blood cells induced by hypochlorous acid (222). In recent years, there has been an increase in interest in foods and plants containing antioxidant properties. The phytochemical compounds present in vegetables and colored fruits with these capacities are vitamins C and E, carotenoids, and flavonoids. These pigments, which are the most important groups of flavonoids, act as acidic compounds because they contain flavylium ions (AH+). As a result, their antioxidant activity is directly influenced by their structure (78, 222–226). According to Aprile et al. (227), the fully matured stage of olive, which is the best harvest time to obtain a table olive, is rich in phenolic compounds, anthocyanins, and other health-promoting nutrients. These compounds are used as antioxidants and anti-inflammatory agents and for preventive measures against several pathological states, such as cardiovascular diseases and tumors (6). In another study by Kim et al. using liquid chromatography and ultra-high-performance liquid chromatography, in combination with electrospray ionization and quadrupole time-of-flight, the antioxidant activity and anthocyanin composition of 12 cultivars of mulberry fruit were investigated and found major anthocyanins like cyanidin-3-O-glucoside and cyanidin-3-O-rutinoside showed significantly higher antioxidant activity (228).

#### Anticarcinogenic effects

In the past, hundreds of compounds including naturally occurring compounds and drugs have been identified as potential chemopreventive agents. Among them, compounds that naturally have the capability of inducing differentiation/apoptosis of cancer cells are the primary anticancer and chemopreventative agents (229). The potential for chemoprevention of cancer has been identified in hundreds of compounds, including drugs and naturally occurring components. According to Liu et al., raspberries inhibit the proliferation of hepatocellular liver carcinoma in a dose-dependent manner (230). Based on their research, Bonesi et al. found that cyanidin-3-glucoside causes apoptosis and cytodifferentiation in several leukemic cell lines (231).

#### Antiobesity and antidiabetic properties

Evidence suggests that a diet low in fat and high in fruits and vegetables reduces the risk of obesity and type-2 diabetes because they are rich in polyphenol compounds, a condition associated with insulin resistance. This condition occurs when insulin fails to effectively stimulate glucose transport in skeletal muscles and fat and fails to suppress hepatic glucose production (232). Anthocyanin has been shown to have lipid-lowering and antioxidant properties as blueberries can inhibit the early inflammatory response in adipose tissue to protect against whole-body insulin resistance and improve glycemia (233). Anthocyanins also play a role in insulin secretion. It protects pancreatic beta cells, from glucose-induced oxidative stress. The anthocyanin extract from colored rice increased the gene expression of genes associated with insulin secretion in rat pancreatic beta cells (234). According to the results, both anthocyanins and anthocyanidins stimulate insulin secretion, and delphinidin-3-glucoside was the most potent compound and significantly affected insulin secretion in comparison to untreated control cells at glucose concentrations of 4 and 10 mol/L (235). Diabetes mellitus (DM) is a chronic metabolic disease characterized by high blood sugar levels (236). Approximately 415 million adults between the ages of 20 and 79 years had diabetes mellitus in 2015, according to the International Diabetes Federation (IDF) (237). Over the past two decades, many efforts have been made to develop natural and less toxic antidiabetic agents. Scientists have been searching for new antidiabetic compounds made from natural sources to minimize side effects (238). Foods that are rich in bioactive compounds such as alkaloids, phenols, flavonoids, saponins, polysaccharides, terpenoids, glycosides, and xanthones can be consumed daily (239). Examples of food containing anthocyanins are berries such as cranberries, chokeberries, blackberries, gooseberries, black grape, bilberries, blueberries, red raspberries, blackcurrants, redcurrants, strawberry, pomegranates, apples, nectarine, peaches, plum radish, and plums; vegetables as red onion, red cabbage, eggplant, and purple potatoes; and seeds as black beans (240).

#### Cardioprotective roles of anthocyanins

The aging of the cardiovascular system involves endothelial dysfunction, intimal hyperplasy, and arterial stiffness, which may lead to arterial arteriosclerosis and atherosclerosis (241). Nutritional interventions are promising approaches to slow down the aging process of the cardiovascular system (241, 242). As a prerequisite for understanding the mechanisms of action of fruits and vegetables, it is imperative to identify bioactive compounds responsible for such beneficial effects and to demonstrate causality using accredited end points. It is also essential to study how such compounds are absorbed, distributed, metabolized, and excreted by healthy humans. Until now, polyphenols are the most promising class of food bioactive compounds found in fruits and vegetables (243, 244). Blueberries are not just a rich source of polyphenols, such as anthocyanin (ACN) but also contain flavonoids, flavonols, and phenolic acids, along with fiber, vitamins, and minerals (245). According to the Nurses' Health Study II, consuming large quantities of blueberries and strawberries, as well as large quantities of anthocyanin (as calculated per food frequency questionnaire), was associated with a lower risk of myocardial infarction (246). Even though these results suggest that anthocyanin intake with blueberries could lower cardiovascular risk, epidemiological data inherently only provide associative evidence, further limited by the lack of biomarkers of intake. Rodriguez et al. in their study suggested that anthocyanin metabolites contribute to healthy cardiovascular aging through mediating biological activities of blueberries and also induced gene expression in a manner that inhibits inflammation and reduces the risk of cardiovascular disease (247). Studies to be conducted in future will enhance our understanding of the mechanisms of action of individual metabolites, establish general structure–function relationships, and identify relevant interactions.

#### Hepatoprotective role of anthocyanins

The hepatotoxic compounds such as carbon tetrachloride (CCl_4_), allyl alcohol, 1-naphthyl isocyanate, and thioacetamide are proven to cause liver tissue necrosis in various areas of the liver in experimental medicine (248). While molecular CCl_4_ is not toxic, its hepatotoxicity arises from the formation of trichloromethyl radicals (^*^CCl_3_) and trichloromethyl peroxide of the radicals (CCl_3_O${}_{2}^{*}$) following liver metabolism (249, 250). Romero et al. studied the effects of CCl_4_ intoxication on protein synthesis (251). Therefore, the total protein content can be considered as a useful index of cellular dysfunction in liver disorders. In a recent study, the ethyl acetate fraction of *Justicia spicigera* was evaluated for its efficiency against CCl_4_-induced liver damage. As indicated by the attenuation of liver function indices and improvement in markers of oxidative stress, ethyl acetate (EA) fraction protects the liver in a pathological condition (252). However, such types of studies have not been undertaken for onion-related anthocyanins and can be investigated in future research projects.

## Conclusion

Recently, anthocyanins from onion are gaining popularity because of diverse health-promoting effects and natural colorants as natural antioxidants. The extraction methods of anthocyanins from onion need optimized conditions because of the sensitivity of anthocyanins. However, the extracted anthocyanins are susceptible to degradation effects by different factors such as pH, light, temperature, enzymes, and oxygen. Although the advanced technology showed a better potential to retain anthocyanins, the use of advanced methods needs further investigation toward industrial application, and due to the high cost of extraction, research could focus on assessing the economic viability of the methods.

Anthocyanins can be helpful in treatment of diseases and show different preventive activities such as antioxidant, antidiabetic, anticancerous, antiobesity, cardioprotective, and hepatoprotective properties. In order to extend the efficiency of anthocyanins to prevent diseases, new directions of research involve the incorporation of anthocyanins into targeted delivery systems that prevents the degradation of anthocyanins, enhances the bioavailability, and provides the stability in different conditions. Thus, different delivery systems have been developed such as dietary fiber-based, emulsions, microcapsules, or liposomes to enhance the bioavailability, and the effects produced by anthocyanins would be stronger. However, future studies are needed to focus on how to reduce the negative impact of each factor that influences the stability of anthocyanins.

## Author contributions

MKS and MS contributed to the organization, writing, and composed of the manuscript. JT and AKP reviewed the manuscript. KK, S, DKY, YT, HR, and MR wrote the sections of manuscript. All authors contributed to the article and approved the submitted version.

## Conflict of interest

The authors declare that the research was conducted in the absence of any commercial or financial relationships that could be construed as a potential conflict of interest.

## Publisher's note

All claims expressed in this article are solely those of the authors and do not necessarily represent those of their affiliated organizations, or those of the publisher, the editors and the reviewers. Any product that may be evaluated in this article, or claim that may be made by its manufacturer, is not guaranteed or endorsed by the publisher.

## Abbreviations

AURKA: aurora kinase A
Akt: protein kinase B
BIRC5: baculoviral IAP repeat containing 5
C4H: cinnamate-4-hydroxylase
4-CL: coumaroyl CoA ligase
COX-2: cyclooxygenase-2
DPPH, 2: 2′-diphenyl-1-picrylhydrazyl
EAE: enzyme-assisted extraction
EGF: Epidermal growth factor
ERK1/2: extracellular signal-regulated kinase 1/2
HPLC: high-performance liquid chromatography
HPLC/DAD-ESI/MS: high-performance liquid chromatography with diode array and mass spectrometry
HPLC- MS: high-performance liquid chromatography–mass spectrometry
HVED: high-voltage electrical discharge
HPLE: high-pressure liquid extraction
iNOS: inducible nitric oxide synthase
MAE: microwave-assisted extraction
NF-*k*B: nuclear factor kappa-light chain-enhancer of activated B cells
ORAC: oxygen radical absorbance capacity methods
PTGS2: prostaglandin-endoperoxide synthase 2
SFE: supercritical fluid extraction
PEFE: pulsed electric fields
UHPLC: ultrahigh-performance liquid chromatography
UAE: ultrasound-assisted extraction.
